# Warner’s New Therapeutic Reference Book

**Published:** 1900-09

**Authors:** 


WARNER'S NEW THERAPEUTIC REFERENCE BOOK.—Regarding this hand-book of therapeutics, we wish to say it is one of the very few guides of its kind now offered students and busy practitioners. As its preface states, it is not intended to teach graduates anything about therapeutics, but it is to be regarded rather as a handy aid to a poor memory. Many exceedingly valuable tables are represented, including the metric table, thermometric equivalents, etc.; valuable tests for various matters, including urinary tests for albumen, sugar, etc., etc.; comparative values of certain foods, a complete dose table of drugs, a list of diseases and their remedies, hints as to indications of pregnancy, recommendations as to post-mortem examinations, etc., etc. The brief mention above gives but a faint idea of the many valuable departments of this new book.

The subjects are interesting, and are written in such a manner as to give a comprehensive idea of what is in the author's mind. "Warner's New Therapeutic Reference Book" must not be confused with "Warner's Therapeutic Reference Book." The latter has been discarded, the new one taking its place. So many new features have been added and the other parts entirely rewritten to a great extent that it may be termed a new book.

It is bound in two styles, one leather at 50 cents, and the other a leatherette at 25 cents per copy, postage prepaid in both instances.

